# A liquid-metal route to direct cathode recycling

**DOI:** 10.1093/nsr/nwag213

**Published:** 2026-04-06

**Authors:** Jiwoong Bae

**Affiliations:** School of Mechanical Engineering, Hanyang University, Republic of Korea

With discarded lithium-ion batteries projected to reach at least 2 million tons per year globally by 2030, battery recycling has emerged as an increasingly urgent technological and environmental challenge. While greener recycling approaches such as deep-eutectic-solvent extraction [1] and contact-electro-catalytic leaching [2] have improved the sustainability of metal recovery, direct recycling remains particularly attractive because it preserves the highest-value fraction of the cell [3]. Much of the embodied economic value of spent batteries resides in cathode-active metals such as Li, Ni, and Co, making their efficient recovery central to any circular battery economy. Yet direct recycling still faces a formidable practical bottleneck: how to separate cathode coatings cleanly from the aluminum (Al) current collector without resorting to energy-intensive heating or corrosive chemical leaching.

**Figure 1.**
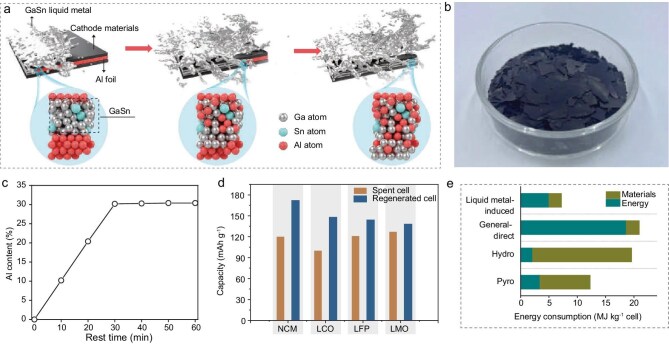
(a) Schematic illustration of liquid-metal-driven separation of the Al current collector from cathode materials. (b) Photograph of recovered spent NCM cathode materials after separation. (c) Al content in the liquid alloy as a function of time during the separation process. (d) Capacity comparison at 0.1 C between spent cells (NCM, LCO, LFP, and LMO) and their regenerated counterparts. (e) Energy consumption of different recycling methods, including liquid-metal-induced recycling, general direct recycling, hydrometallurgy (Hydro) and pyrometallurgy (Pyro). Reproduced from ref. [4] with permission.

In a recent study published in *National Science Review*, Prof. Yu Ding and collaborators reported a highly efficient solution based on GaSn liquid metals [4]. As illustrated in Fig. 1a, the liquid alloy selectively targets the Al foil rather than the cathode itself. Upon contact, liquid metals rapidly wet the Al surface, disrupt its native passivation layer, and diffuse along grain boundaries. Supported by density functional theory calculations revealing a high binding energy between liquid metals and the Al(110) facet, this thermodynamically driven permeation weakens intergranular cohesion, dismantles the current collector, and ultimately nondestructively liberates the active layer. The process proceeds at room temperature and achieves nearly complete detachment within 30 min, with a separation efficiency of about 99.4%. Importantly, the recovered cathode materials are essentially free of residual Al (Fig. 1b), underscoring the high selectivity of the method.

The separation mechanism is supported by the evolution of Al content in the liquid metal, which rises rapidly and plateaus within 30 min (Fig. 1c), consistent with fast Al dissolution and foil disintegration. Equally notable is what the liquid metal does not dissolve: transition-metal contamination in the regenerated alloy remains virtually undetectable (below 0.002 wt.% for Ni, Co, Mn, and Fe). This is a critical advantage for direct recycling, since the economic and functional viability of this approach hinges on preserving the intrinsic crystal structure of the active materials [5]. After a subsequent direct annealing process in the presence of lithium salts, regenerated electrodes including LiNi_1/3_Co_1/3_Mn_1/3_O_2_ (NCM), LiCoO_2_ (LCO), LiFePO_4_ (LFP), and LiMn_2_O_4_ (LMO) deliver high reversible capacities (Fig. 1d), showing that the process is not merely an effective means of delamination, but also a regeneration-compatible route for recovering functional cathodes.

The broader significance of this work lies in how it fundamentally redefines the energy landscape of battery recycling. Conventional routes rely on heat, acids, or both, while even direct recycling can involve substantial energetic burdens upstream and during separation. By contrast, the liquid-metal route reduces the problem to a selective physical-chemical interfacial reaction. This simplicity translates into a striking energetic advantage: the total energy consumption is only 7.18 MJ kg^−1^ cell, significantly lower than general direct recycling (20.92 MJ kg^−1^), hydrometallurgy (19.57 MJ kg^−1^), and pyrometallurgy (12.26 MJ kg^−1^), as highlighted in Fig. 1e. Moreover, the GaSn liquid metal can be instantly regenerated through a spontaneous reaction with water to remove dissolved aluminum, enabling closed-loop Al recovery while yielding high-purity H_2_ gas as a value-added, clean-energy byproduct without generating harmful emissions.

Overall, this work demonstrates how harnessing selective interfacial chemistry can simplify cathode recycling while retaining the value of both the cathode and the current collector. While questions regarding industrial scale-up and techno-economic optimization at the gigawatt-hour scale remain, this study points toward a more circular route to battery recycling, in which selective removal of the aluminum current collector preserves the compositional integrity of cathodes while simultaneously enabling sustainable material recovery and on-demand H_2_ generation.
